# Evaluating responses by ChatGPT to farmers’ questions on irrigated lowland rice cultivation in Nigeria

**DOI:** 10.1038/s41598-024-53916-1

**Published:** 2024-02-10

**Authors:** Ali Ibrahim, Kalimuthu Senthilkumar, Kazuki Saito

**Affiliations:** 1 Africa Rice Center (AfricaRice), PMB 82, Abuja, 901101 Nigeria; 2 Faculté d’Agronomie, Université Abdou Moumouni, B.P. 10960, Niamey, Niger; 3 Africa Rice Center (AfricaRice), B.P. 1690, 101 Antananarivo, Madagascar; 4 Africa Rice Center (AfricaRice), 01 B.P. 2551, Bouaké 01, Côte d’Ivoire; 5 International Rice Research Institute (IRRI), DAPO Box 7777, 1301 Metro Manila, Philippines

**Keywords:** Ecology, Software

## Abstract

The limited number of agricultural extension agents (EAs) in sub-Saharan Africa limits farmers’ access to extension services. Artificial intelligence (AI) assistants could potentially aid in providing answers to farmers’ questions. The objective of this study was to evaluate the ability of an AI chatbot assistant (ChatGPT) to provide quality responses to farmers’ questions. We compiled a list of 32 questions related to irrigated rice cultivation from farmers in Kano State, Nigeria. Six EAs from the state were randomly selected to answer these questions. Their answers, along with those of ChatGPT, were assessed by four evaluators in terms of quality and local relevancy. Overall, chatbot responses were rated significantly higher quality than EAs’ responses. Chatbot responses received the best score nearly six times as often as the EAs’ (40% vs. 7%). The evaluators preferred chatbot responses to EAs in 78% of cases. The topics for which the chatbot responses received poorer scores than those by EAs included planting time, seed rate, and fertilizer application rate and timing. In conclusion, while the chatbot could offer an alternative source for providing agricultural advisory services to farmers, incorporating site-specific input rate-and-timing agronomic practices into AI assistants is critical for their direct use by farmers.

## Introduction

In sub-Saharan Africa (SSA), rice productivity is often low due to sub-optimal crop management practices by smallholder farmers^1–3^. Farmers have limited access to agricultural extension services due to the limited number of extension agents (EAs), which results in many rice farmers not having access to updated advice for rice production^4,5^. Furthermore, within rural socio-cultural systems, EAs often do not effectively reach women farmers. In some areas in SSA, women are negatively affected by socio-cultural and religious constraints, which forbid them from communicating freely with men outside their families^5^. A wide variety of technology dissemination and scaling tools (rural radio, videos, etc.) have been developed and used to reach women farmers^6,7^. A dissemination approach in which women service providers reach women farmers has been also proposed for providing field-specific recommendations to farmers, which requires service providers to have digital technologies (smartphone, tablet)^5^. While further efforts are needed to improve access to electricity and internet to aid the adoption of digital extension services in the rural agrarian communities in SSA, recent development of artificial intelligence (AI) assistance is an unexplored resource for addressing challenges farmers face. One such platform, ChatGPT, represents a new generation of AI technologies driven by advances in large language models^8^. A recent study on health care reported that although the system was not developed to provide health care, the chatbot responses were preferred over physician responses and rated significantly higher for both quality and empathy^9^. However, its ability to help address farmers’ questions on rice cultivation in SSA is unexplored.

Therefore, the objective of this study was to evaluate the ability of an AI chatbot assistant (ChatGPT) to provide quality responses to farmers’ questions on rice production. We tested ChatGPT’s ability to respond with high-quality answers to farmers’ questions, by comparing the chatbot responses with EAs’ responses to questions in Kano State, one of major rice producing areas in northern Nigeria^10,11^.

## Results

**Figure 1:**
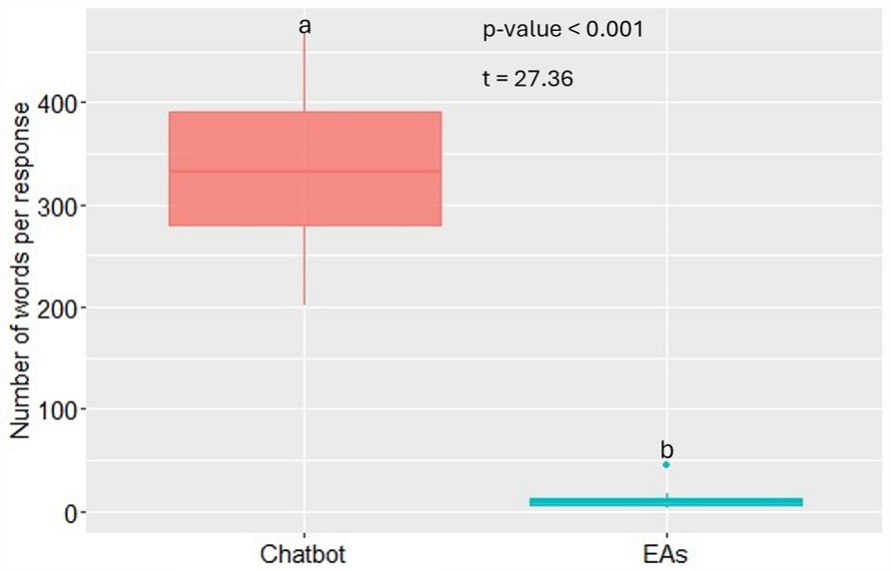
Number of words per response authored by extension agents (EAs) and chatbot. As there was no difference in number of words per response by EAs without and with extension materials, data from both were combined. Different letter indicates significant difference (*P* < 0.001).

Table 1 shows questions related to rice production, which are based on answers from 107 interviewed farmers about questions they want to ask EAs for improving their rice production. Popular questions mentioned by farmers were on types of inputs (variety, fertilizer, herbicide). In terms of number of questions in each intervention area, crop establishment, insect and disease management, and weed management had most (5, 5, and 4, respectively). Examples of EAs’ and chatbot responses to questions (nos. 1–3) are shown in Table 2. Mean chatbot responses were significantly longer (335 [202–468] words) than both EAs’ responses with and without extension materials, which had no difference (10 [2–45]) (Fig. 1).

**Table 1:** List of questions used for this study, and the target area in terms of agronomic practice. Questions are in order of number of farmers giving the same or similar questions (most to fewest).

Question no	Intervention area	Question	No. farmers
1	Variety	Which rice variety is recommended for irrigated rice cultivation in Kano State, Nigeria?	49
2	Nutrient management	Which types of fertilizers are recommended for irrigated rice cultivation in Kano State, Nigeria?	33
3	Weed management	Which types of herbicides are recommended for irrigated rice cultivation in Kano State, Nigeria?	33
4	General agronomy	Please provide with recommended rice production practices for improving yield of irrigated lowland rice in Kano State, Nigeria	21
5	Insect & disease management	Which types of pesticides are recommended for irrigated rice cultivation in Kano State, Nigeria?	15
6	Insect & disease management	Please provide with recommendation for stem borer control for irrigated lowland rice in Kano State, Nigeria	13
7	Seed	Please tell us how to access good rice seed in Kano State, Nigeria	11
8	Weed management	Please provide with recommended weed management practices for irrigated lowland rice cultivation in Kano State, Nigeria	11
9	Soil	What type of soil is the best suited to irrigated lowland rice production in Kano State, Nigeria?	11
10	Seed	What is the recommended quantity of seeds per hectare for direct seeded rice in Kano State, Nigeria?	10
11	Seed	What is the recommended quantity of seeds per hectare for transplanted rice in Kano State, Nigeria?	10
12	Crop establishment	When is recommended rice planting time in direct seeded rice in irrigated lowland for both wet and dry seasons in Kano State, Nigeria?	8
13	Crop establishment	When is recommended rice planting time in transplanted rice in irrigated lowland for both wet and dry seasons in Kano State, Nigeria?	9
14	Finance service	Is there any financial support for purchasing chemical inputs for irrigated lowland rice cultivation in Kano State, Nigeria?	6
15	Crop establishment	What is the best rice establishment method for irrigated lowland rice in Kano State, Nigeria?	6
16	Nutrient management	Please provide with recommended fertilizer application practices for irrigated lowland rice cultivation in Kano State, Nigeria	4
17	Nutrient management	Are there any organic inputs available for irrigated lowland rice cultivation in Kano State, Nigeria?	4
18	Bird control	What are recommended control measures for birds in irrigated lowland rice field in Kano State, Nigeria?	4
19	Crop establishment	What is recommended timing for transplanting rice seedlings from nursery for irrigated lowland rice in Kano State, Nigeria?	3
20	Abiotic stress	How to alleviate flooding problem in lowland rice fields in Kano State, Nigeria?	3
21	General agronomy	What are ways of reducing the cost of irrigated lowland rice production in Kano State, Nigeria?	3
22	Weed management	How to identify dosage for herbicide application for irrigated lowland rice cultivation in Kano State, Nigeria?	3
23	Crop establishment	What is recommended number of seedlings to transplant rice per hill for irrigated lowland rice in Kano State, Nigeria?	2
24	Variety	Are there weed competitive varieties suitable to irrigated lowland rice production in Kano State, Nigeria?	2
25	Variety	Are there early maturing rice varieties suitable to irrigated lowland rice production in Kano State, Nigeria?	2
26	Water management	Please describe the optimum water depth for irrigated lowland rice cultivation in Kano State, Nigeria	2
27	Insect & disease management	How to identify dosage for pesticide application for irrigated lowland rice cultivation in Kano State, Nigeria?	2
28	Weed management	When is recommended timing of herbicide application for irrigated lowland rice cultivation in Kano State, Nigeria?	2
29	Insect & disease management	How to control rust in rice plants in Kano State, Nigeria?	2
30	Insect & disease management	When is recommended timing of pesticide application for irrigated lowland rice cultivation in Kano State, Nigeria?	1
31	Abiotic stress	How to manage salinity in irrigated lowland rice cultivation in Kano State, Nigeria?	1
32	Land preparation	What are recommended land preparation practices for irrigated lowland rice production in Kano State, Nigeria?	1

Total number of farmers are higher than 107 farmers interviewed, as farmers gave up to five questions.

**Table 2:** Example of extension agents’ and chatbot responses to questions related to rice production in Kano State, Nigeria.

Question no	Extension agents	Chatbot
1	Faro 44, Faro 52, Faro 57, Faro 66 and Faro 67	In Kano State, Nigeria, the recommended rice variety for irrigated rice cultivation may vary based on several factors such as local agroecological conditions, market demand, and farmers’ preferences. However, as of my knowledge cutoff in September 2021, some of the rice varieties commonly recommended for irrigated rice cultivation in Nigeria include: Faro 44: This is a popular variety developed by the National Cereal Research Institute (NCRI). Faro 44 is known for its high yield potential, good grain quality, and resistance to diseases such as rice blast Faro 52: Another variety developed by NCRI, Faro 52 is suitable for irrigated conditions. It is known for its good yield, grain quality, and resistance to diseases and pests FARO 60: This variety is well-suited for irrigated rice farming and is popular among farmers in Nigeria. It has good yield potential, high grain quality, and resistance to diseases and pests It’s important to note that rice breeding programs continue to develop new and improved varieties, and the recommended varieties may have changed since my knowledge cutoff in 2021. Therefore, it is advisable to consult with local agricultural extension services, research institutes, or agricultural experts in Kano State for the most up-to-date and region-specific recommendations on rice varieties for irrigated cultivation
2	NPK 20:10:10, NPK 20:10:5, NPK 15:15:15, urea 46:0:0, DAP, CALUR (slow release)	For irrigated rice cultivation in Kano State, Nigeria, the recommended types of fertilizers can vary depending on the specific soil conditions, nutrient deficiencies, and the growth stage of the rice crop. However, some commonly used fertilizers for rice cultivation include: NPK fertilizers: NPK stands for nitrogen (N), phosphorus (P), and potassium (K), which are essential macronutrients for rice plants. Balanced NPK fertilizers with a ratio appropriate for rice, such as 15–15-15 or 20–10-10, are commonly used. These fertilizers help provide the necessary nutrients for healthy plant growth, root development, and grain formation Urea: Urea is a common nitrogen fertilizer used in rice cultivation. Nitrogen is particularly important for rice as it is a major component of chlorophyll, which is essential for photosynthesis. Urea is usually applied in split doses during different growth stages of the rice crop Single Superphosphate (SSP) or Triple Superphosphate (TSP): Superphosphate fertilizers are good sources of phosphorus. Phosphorus plays a crucial role in root development, flowering, and seed formation. SSP or TSP fertilizers can be used to address phosphorus deficiencies in the soil Muriate of Potash (MOP): Potassium is essential for overall plant health, water regulation, disease resistance, and grain quality. Muriate of Potash is a common potassium fertilizer used in rice cultivation to ensure an adequate supply of potassium It is important to note that the fertilizer requirements may vary depending on soil nutrient levels, crop rotation practices, and other factors. Conducting a soil test prior to planting can provide valuable information about the specific nutrient needs of your rice crop. It is recommended to consult with local agricultural extension services, soil experts, or agronomists for precise fertilizer recommendations based on the specific conditions in Kano State and the latest agricultural practices
3	Glyphosate = vegetation clearance; Butachlor = pre-emergence; Propanil + 2,4D = post emergence	For irrigated rice cultivation in Kano State, Nigeria, several herbicides are commonly recommended to manage weeds effectively. The choice of herbicide depends on the specific weed species present, the growth stage of the rice crop, and other factors. Here are some commonly used herbicides in rice cultivation: Pre-emergence herbicides: Pendimethalin: This herbicide is effective against grassy and broadleaf weeds. It is commonly used as a pre-emergence herbicide to control weeds before they emerge from the soil Oxadiazon: It provides pre-emergence control of a wide range of annual grasses and broadleaf weeds in rice fields Post-emergence herbicides: 2,4-D: This herbicide is effective against broadleaf weeds. It is commonly used as a post-emergence herbicide in rice crops Bispyribac-sodium: It is a post-emergence herbicide effective against grassy weeds, including some problematic rice weeds like *Echinochloa* spp. (barnyardgrass) Cyhalofop-butyl: This herbicide is used to control grassy weeds in rice fields, especially against *Echinochloa* spp. Integrated weed management (IWM): Integrated weed management involves using a combination of different control methods, including cultural, mechanical, and chemical methods. It focuses on sustainable weed management and reducing herbicide reliance. Some cultural practices used in IWM for rice cultivation include proper land preparation, crop rotation, and water management It is important to note that herbicide selection should be based on local weed species, specific field conditions, and following recommended application rates and safety precautions. Additionally, it is advisable to consult with local agricultural extension services, agricultural experts, or research institutes in Kano State for the most up-to-date and region-specific recommendations on herbicides for irrigated rice cultivation. They can provide guidance on appropriate herbicide options and application practices while considering factors such as herbicide resistance management and environmental sustainability

On average over 32 questions, evaluators rated chatbot responses significantly higher quality than responses by EAs without and with extension materials by 19 and 15% (*P* < 0.01) (Table 3). The mean rating for chatbot responses corresponded to a “good” response (3.8), whereas those for EAs’ responses without and with extension materials corresponded to an acceptable response (3.2 and 3.3, respectively). There was no significant difference in scores between EAs’ responses without and with extension materials. The Pearson correlation coefficient between scores of responses by EAs without and with extension materials was positive and significant (r = 0.71, *P* < 0.01). The correlation coefficients between scores of responses by chatbot and EAs without and with extension material were not significant (r = − 0.13, *P* > 0.05; r = − 0.15, *P* > 0.05).

**Table 3:** Mean scores of responses by extension agents (EAs) with and without extension materials and chatbot to 32 questions.

Question no	EA without extension materials	EAs with extension materials	Chatbot
1	3.3a	4.0a	3.8a
2	3.8a	3.4a	4.0a
3	4.0a	4.5a	4.0a
4	3.3b	3.5ab	4.5a
5	3.3b	3.3b	4.5a
6	3.0b	2.8b	4.8a
7	3.3b	3.5b	4.8a
8	2.8b	3.0b	4.8a
9	4.0a	3.8a	4.8a
10	3.8a	4.0a	4.0a
11	3.5a	3.5a	2.5b
12	3.8ab	4.0a	2.5b
13	2.0a	2.0a	3.0a
14	2.8a	3.0a	2.8a
15	3.5a	3.8a	3.0a
16	3.0a	2.8a	2.3a
17	3.3a	3.5a	3.8a
18	3.5a	3.5a	4.0a
19	2.8b	3.ab	3.8a
20	3.3a	3.5a	3.5a
21	2.3b	2.3b	4.5a
22	2.5b	2.8b	4.8a
23	4.0a	4.3a	3.0b
24	2.5a	2.8a	3.3a
25	3.3a	3.5a	3.8a
26	4.3a	4.5a	4.0a
27	3.3b	3.5b	4.3a
28	3.0a	3.0a	3.5a
29	2.5b	2.8b	4.5a
30	2.8a	2.8a	2.8a
31	2.8b	2.8b	4.8a
32	3.3b	3.3b	4.8a
Av	3.2b	3.3b	3.8a

Within a row, different letters indicate statistically significant differences (*P* ≤ 0.05).

The proportion of responses rated very good quality (5; range between 1 and 5) was significantly higher (*p* < 0.05) for chatbot responses than for those of EAs without and with extension materials (Table 4). The chatbot achieved the best score nearly six times as often as EAs (40% vs. 6% and 8%). In contrast, the proportion of responses rated acceptable was significantly lower for chatbot compared to EAs without and with extension materials (18% vs. 51% and 46%; Table 4). There was no significant difference in the number of responses rated poor and very poor between the chatbot and EAs without and with extension materials (Table 4).

**Table 4:** Distribution (%) of evaluators’ scores on responses by extension agents (EAs) with and without extension materials and chatbot to 32 questions.

	Very good (Score 5)	Good (Score 4)	Acceptable (Score 3)	Poor (Score 2)	Very poor (Score 1)
EAs without extension materials	2 (6)b	8 (26)a	16 (51)a	5 (15)a	1 (2)a
EAs with extension materials	3 (8)b	11 (34)a	14 (46)a	3 (9)a	1 (3)a
ChatGPT	13 (40)a	8 (24)a	5 (18)b	5 (15)a	1 (3)a
Pearson chi-squared (*p*-value)	< 0.001***	0.314ns	< 0.001***	0.327ns	0.67ns

Evaluators judged “the quality of information provided” with scores as very poor, poor, acceptable, good, or very good. Within a column, different letters indicate statistically significant differences (*P* ≤ 0.05).

As the scores recorded vary across evaluators, we made an average for each score. The percent can be different depending on the average; for example, if the average number of responses with a score of 3 is 7.75 and 8.25, the percent is 24 and 26, respectively. *** statistical significance at and 0.1% (*P* < 0.001) level; ns, not significant. Values in the brackets denote the value in percentage.

**Figure 2:**
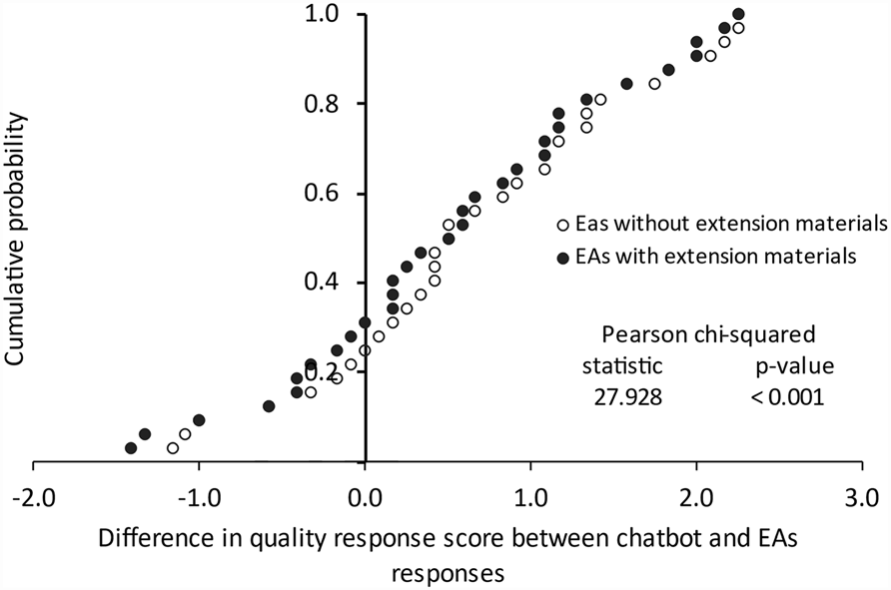
Cumulative probability of the difference in score between responses authored by extension agents (EAs) without and with extension material and chatbot. Response scoring options had a 1–5 scale, where higher values indicated greater quality.

Across the 32 questions, the evaluators preferred the chatbot response over the responses by EAs without and with extension materials for 78% and 69%, respectively (Fig. 2). When we looked at the responses where the chatbot had lower scores than those authored by EAs (questions 11, 12, and 23 in Tables 1 and 3) and having lower score than 3 (14 and 16), we found that the chatbot provided inaccurate information (Table 5)—i.e., the chatbot-recommended seed rate was too high (11); planting time was not correct in dry season (12); financial services were not available (14); soil testing was not recommended (16); recommended number of seedlings per hill was different but should not be different between the two seasons (23).

**Table 5:** Responses authored by extension agents (example) and chatbot (summary only), for questions where chatbot responses had lower scores than those of extension agents (Tables 1 and 3).

No	Question	Extension agents (example)	Chatbot (Summary only)
11	What is the recommended quantity of seeds per hectare for transplanted rice in Kano State, Nigeria?	35–40 kg/ha	The recommended seeding rate for transplanted rice ranges from 80 to 120 kg/ha
12	When is recommended rice planting time in direct seeded rice in irrigated lowland for both wet and dry seasons in Kano State, Nigeria?	Dry season: 3rd week of January to 2nd week of February; wet season: 2nd week of June to 2nd week of July when rain has fully established	For wet season, the recommended planting time is around May to June For dry season, the planting time generally starts in November to December
14	Is there any financial support for purchasing chemical inputs for irrigated lowland rice cultivation in Kano State, Nigeria?	No	There are various government programs and financial support initiatives in Nigeria aimed at providing assistance to farmers, including those involved in irrigated lowland rice cultivation
• 16	• Please provide with recommended fertilizer application practices for irrigated lowland rice cultivation in Kano State, Nigeria	• Per hectare, use 6 × 50 kg of fertilizer (4 bags of NPK as application and 2 bags of urea as second application)	• Conduct soil testing before planting and follow the soil test recommendations for the application of nutrients • Apply a portion of the recommended fertilizer dose as a basal application before planting or during land preparation • The first top-dressing application can be done around 10–14 days after transplanting or when direct-seeded rice reaches the 2–3 leaf stage • Apply the majority of the nitrogen fertilizer during the vegetative stage (around 20–30 days after transplanting) • Apply phosphorus and potassium fertilizers as recommended during the basal application and consider incorporating them into the soil during land preparation
23	What is recommended number of seedlings to transplant rice per hill for irrigated lowland rice in Kano State, Nigeria?	2–3 seedlings per hill	For irrigated lowland rice in the wet season, it is typically recommended to transplant 2 to 3 seedlings per hill Transplanting 3 to 4 seedlings per hill is often recommended in the dry season

After reviewing the chatbot responses, five out of the six EAs who had answered the 32 questions indicated that the chatbot provided relevant answers on rice cultivation and could be used as a tool for EAs to provide farmers with advice (Table 6). All EAs rated the chatbot responses better than their own answers to the questions, and were willing to use chatbot in the future to get the required information to assist farmers.

**Table 6:** Responses of the six extension agents after reviewing the chatbot responses.

Question	Answer
Did the chatbot provide relevant answers on rice cultivation in Kano?	Yes (5) Neither yes nor no (1)
Do you think that the chatbot can be used as a tool for extension agents to provide farmers with advices?	Yes (5) Neither yes nor no (1)
How would you rate the chatbot’s responses compared to your own answers to the questions?	Better (all)
Will you use chatbot in the future to get the required information to assist farmers?	Yes (all)

## Discussion

While chatbot responses were much longer than EAs’ responses, the evaluators preferred chatbot-generated responses over those by EAs even when the latter had extension materials. In fact, having extension materials did not significantly improve quality scores and the scores were highly correlated between responses by EAs with and without extension materials. The chatbot is programmed to provide detailed and comprehensive responses, whereas EAs may provide more concise and practical advice based on their experience. However, the study also found that the evaluators preferred chatbot responses over those provided by EAs, even when the latter had extension materials. Although the evaluators valued the detailed and comprehensive information provided by the chatbot, farmers might have different opinions from them. Longer answers by the chatbot could potentially overwhelm farmers with too much information. Further evaluation by farmers is needed, if the chatbot is directly used by farmers.

This result also confirmed a recent study on health^9^, which reported that chatbot responses were preferred over physician responses and rated significantly higher for both quality and empathy. The results from this study suggest that a chatbot might become a useful source of information for advising farmers who have limited access to EAs. However, there was no relationship between scores on the responses by the chatbot and EAs and the chatbot provided inaccurate information related to planting time, seed rate, and fertilizer application rate and timing and that message should be made known to rural farmers. Our result supports the paper on large language models (LLMs) and agricultural extension services^12^ which proposed an idealized LLM design process with human experts in the loop. Consequently, direct use of this tool by farmers is not recommended at present. Instead of direct use, chatbot could assist EAs when giving advice to farmers by drafting a message based on farmers’ questions. Such an AI-assisted approach could save EAs’ time, enabling them to reach more farmers. Furthermore, EAs could also improve their overall communication skills by reviewing and modifying AI-written drafts. Consequently, further research is needed to evaluate how an AI assistant will enhance EAs responding to farmers’ questions and improve their skills and knowledge.

For direct use by farmers, this study highlights the importance of ensuring that the chatbot is programmed with accurate and up-to-date information and that their responses are regularly reviewed and updated by experts in the field. This could involve ensuring that the AI assistant technologies are tailored to the needs and context of the farmers, providing practical and actionable advice, and ensuring that the AI assistant technologies are developed and implemented in a way that is transparent, accountable, and responsive to the needs and concerns of farmers. By addressing these challenges, farmers could directly benefit from AI assistant technologies. Further research is also needed to evaluate farmers’ perception of advisories provided by AI assistant technologies, changes in farmers’ practices after receiving advisories, and their target impact area (e.g., productivity, resource use efficiency, soil health)^13^.

## Methods

In June 2023, we conducted interviews with farmers who grow rice in irrigated conditions in Kano State, northern Nigeria. Seventeen women and 90 men farmers were randomly selected from 4032 farmers who had participated in an on-farm survey the previous year (unpublished data) and were asked about questions they want to ask EAs for improving their rice production. Each farmer provided up to five questions. After compiling all the questions, similar questions were merged. We also removed some questions that were not relevant for irrigated rice production (e.g., drought-tolerant varieties). We modified questions to make sure that we consistently included information on location and rice production system and protected farmers’ identities. Table 1 shows the list of 32 questions used in this study, which covered a wide range of agronomic interventions including seed, variety, land preparation, crop establishment method, and management of nutrient, water, weeds, and insects and disease.

On August 10, 2023, the full text of the questions (Table 1) was put into a fresh chatbot session^8^ free of prior questions that could bias the results, and the chatbot response was saved in a Word file.

Six EAs were nominated from an agricultural extension office in Kano based on their expertise and knowledge of rice cultivation practices. To protect EAs’ identities, we do not specify names of the organizations in this paper. Three of the agents were women. None of them had used a chatbot for their extension services before. They were divided into two groups. One group (three agents) used extension materials for answering questions, while the other group did not. They wrote answers to questions on paper in their offices under the supervision of enumerators. The number of words in the responses by EAs with/without extension materials and the chatbot were counted. After EAs completed their responses, they reviewed the chatbot responses and were then asked about its potential use.

After all responses from the six EAs and the chatbot were compiled, for each question, order of the seven answers were randomized. So that, the order can be different from one question to another. Then, we labeled 1 to 7 in each question to blind evaluators to the identity of the responders. We eliminated information that could be used to identify respondents’ identity by evaluators (for a chatbot, we eliminated statements such as “I’m an artificial intelligence”). All the responses were evaluated by four local rice experts—two from research organizations and others from public extension agencies having good knowledge of local rice production. The evaluators were asked to judge the quality of the responses in terms of local relevance using Likert scales (1, very poor; 2, poor; 3, acceptable; 4, good; and 5, very good).

Scores were averaged across evaluators for each question. This method is used when there is no ground truthing in the outcome being studied, and the evaluated outcomes themselves are inherently subjective. Thus, the mean score reflects evaluator consensus, and disagreements (or inherent ambiguity, uncertainty) between evaluators is reflected in the score variance. Thus, analysis of variance (ANOVA) was conducted to assess difference in the quality score of EAs with and and without extension materials responses to ChatGPT responses. The chi-squared test was applied to identify significant differences between evaluators’ scores on responses by extension agents (EAs) with and without extension materials and chatbot. For the chi-squared test, the null hypothesis states that there is no significant difference between the evaluators’ scores, whereas the alternative hypothesis states that these scores differ. We employed a t-test to compare the difference in the number of words in EAs and chatbot responses because the number of words in EAs with and without content is similar. Shapiro and Bartlett tests were used before ANOVA and t-tests to ensure that the data had a normal distribution and was homogeneous in terms of variance. Mean separation was done using the Tukey HDS approach. Pearson correlation between scores of the responses of EAs and the chatbot was performed. All statistical analyses were performed in R statistical software, version 4.3.1^14^.

The distribution of the expert assessment of the responses is presented in Fig. 2. We report the percentage of questions for which the chatbot response was preferred and identified the questions in which the chatbot responses had lower scores than those of EAs.

### Ethical approval

The authors confirm that all methods were carried out in accordance with relevant guidelines and regulations. The authors confirm that all experimental protocols were approved by Africa Rice Center Scientific Committee. The authors confirm that informed consent was obtained from all subjects involved in this study.

## Data Availability

Data will be made available on request. Email could be sent to Dr. Ali Ibrahim (i.ali@cgiar.org).
